# Evaluating the Evolutionary Origins of Unexpected Character Distributions within the Bacterial Planctomycetes-Verrucomicrobia-Chlamydiae Superphylum

**DOI:** 10.3389/fmicb.2012.00401

**Published:** 2012-11-23

**Authors:** A. Budd, D. P. Devos

**Affiliations:** ^1^European Molecular Biology LaboratoryHeidelberg, Germany

**Keywords:** PVC superphylum, lateral gene transfer, LGT, gene loss, gene duplication, phylogenetic estimation errors

## Abstract

Recently, several characters that are absent from most bacteria, but which are found in many eukaryotes or archaea, have been identified within the bacterial Planctomycetes-Verrucomicrobia-Chlamydiae (PVC) superphylum. Hypotheses of the evolutionary history of such characters are commonly based on the inference of phylogenies of gene or protein families associated with the traits, estimated from multiple sequence alignments (MSAs). So far, studies of this kind have focused on the distribution of (i) two genes involved in the synthesis of sterol, (ii) tubulin genes, and (iii) c1 transfer genes. In many cases, these analyses have concluded that horizontal gene transfer (HGT) is likely to have played a role in shaping the taxonomic distribution of these gene families. In this article, we describe several issues with the inference of HGT from such analyses, in particular concerning the considerable uncertainty associated with our estimation of both gene family phylogenies (especially those containing ancient lineage divergences) and the Tree of Life (ToL), and the need for wider use and further development of explicit probabilistic models to compare hypotheses of vertical and horizontal genetic transmission. We suggest that data which is often taken as evidence for the occurrence of ancient HGT events may not be as convincing as is commonly described, and consideration of alternative theories is recommended. While focusing on analyses including PVCs, this discussion is also relevant for inferences of HGT involving other groups of organisms.

## The PVC Superphylum

The Planctomycetes-Verrucomicrobia-Chlamydiae (PVC) superphylum is an assemblage of bacterial phyla which is consistently recovered as a monophyletic group in trees using a range of different phylogeny estimation methods and data (Wagner and Horn, 2006; Pol et al., 2007; Hou et al., 2008; Pilhofer et al., 2008; Kamneva et al., 2010). It includes the Planctomycetes (Fuerst and Sagulenko, 2011), the Verrucomicrobia, the Chlamydiae, the Poribacteria, the Lentisphaerae, and the OP3 candidate phyla (consisting only of uncultured organisms), along with several other groups. A range of characters that were previously either considered absent or rare amongst bacteria, but which are common or ubiquitous in archaea or eukaryotes, have recently been identified in some PVC members (Table 1, Appendix). These include, for example, the presence of membrane coat (MC)-like proteins and condensed DNA. A list of such features has been described elsewhere (Devos and Reynaud, 2010; Reynaud and Devos, 2011). In this article we review several analyses of the taxonomic distribution of those characters.

**Table 1:** **Summary of previously published analyses of unexpected taxonomic character distributions**.

Reference	Character	Results	Conclusion
Pearson et al. (2003)	Sterol synthesis	Basal branching	HGT
Chen et al. (2007)	Sterol synthesis	Basal branching	HGT
Desmond and Gribaldo (2009)	Sterol synthesis	Basal and internal branching	HGT or VGT
Frickey and Kannenberg (2009)	Sterol synthesis	Intermediate	HGT or VGT
Jenkins et al. (2002)	Tubulin	Basal branching	VGT
Pilhofer et al. (2011)	Tubulin	Basal branching	VGT
Chistoserdova et al. (2004)	Methanogenesis	Intermediate	VGT
Bauer et al. (2004)	Methanogenesis	Intermediate	HGT or VGT
Vorholt et al. (2005)	Methylotrophy	Intermediate	VGT
Kalyuzhnaya et al. (2005)	Methylotrophy	Basal branching	HGT
Op den Camp et al. (2009)	Methanotrophy	Intermediate	VGT
Dunfield et al. (2007)	Methanotrophy	Internal branching	VGT
Khadem et al. (2011)	Rubisco	Intermediate	VGT

*For each publication (citation provided in the “Reference” column), the table indicates the character analyzed, a summary of the phylogenetic relationships (“Results”), and conclusions reached in the reference concerning the evolutionary origins of the unexpected character distributions (“Conclusions”). In the “Results” column, according to the trees presented in the publication: “Basal Branching” indicates that the most recent lineage (i.e., branch) common to the ancestry of sequences from both PVC and other organisms is near the root of the tree; “Internal Branching” indicates that one or more PVC genes are more closely related to a specific subset of eukaryotes than they are to others (i.e., the PVC genes emerged from “within” the eukaryotes); “Intermediate” indicates that the unrooted phylogenies contain three clusters (i.e., clans) of sequences (PVC, non-PVC bacteria, and others). In the “Conclusions” column: “HGT” indicates that the most likely scenario for the evolution of the gene family was inferred to involve some instance of HGT; “VGT” (vertical gene transfer) indicates that the most likely scenario for the evolution of the gene family was inferred NOT to involve HGT; “HGT or VGT” indicates that the authors considered the evidence to provide similar levels of support for the HGT and VGT alternatives*.

## Investigating the Evolutionary Basis for Character Distributions Using Gene and Organism Phylogenies

**Figure 1:**
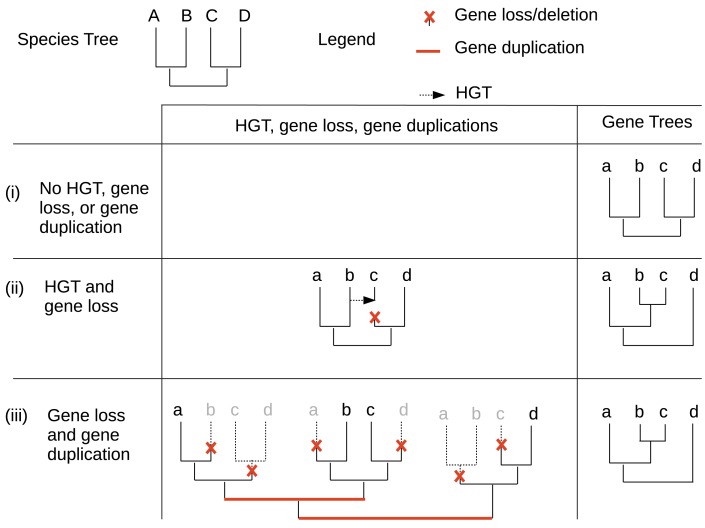
**Influence of HGT, gene loss, and gene duplication on phylogenetic and taxonomic distribution of gene families**. Three different hypothetical evolutionary scenarios are shown for gene family evolution within a simple four-taxon species tree: (i) in the absence of HGT, gene loss, or gene duplication, the gene tree has the same topology as the species tree (ii) a combination of HGT and gene loss yields a gene tree with a different topology to the species tree (iii) a combination of gene loss and gene duplication yields a gene tree with a different topology to the species tree, with the same gene tree topology as for scenario (ii).

The taxonomic distribution of a heritable character can be shaped by many different genetic events. These include vertical or horizontal inheritance of genes responsible for the phenotype, loss or duplication of these genes, and independent or convergent acquisition of a phenotype. Phylogenetic trees estimated from molecular data, i.e., from multiple sequence alignments (MSAs), play a key role in our attempts to estimate the contribution of those mechanisms to the evolution of any character. To use MSAs in this way, we need to know (some of) the genes or proteins responsible for expressing the character of interest, and to be able to estimate reasonably accurate MSAs for the relevant sequences. The precision of the phylogenies estimated from these MSAs is assessed by branch support values such as non-parametric bootstrap values; note, however, that such values represent the precision (i.e. sampling error) with which the topology of these phylogenetic trees are estimated, not the accuracy of these estimates. The resulting gene or protein tree is then compared to the corresponding organism tree. The phylogenetic tree describing the evolution of a gene family which does not experience any gene loss, duplication, incomplete lineage sorting, intra-gene recombination, or horizontal gene transfer (HGT), and which only evolves via a process of point mutation, will have the same topology as that of the corresponding organism (Figure 1). In contrast, where the evolution of a gene family does involve gene loss, duplication, incomplete lineage sorting, intra-gene recombination, or HGT, the gene tree topology may no longer correspond to the organism tree topology. For this reason, the observation of differences between the topologies of gene and organism trees has been used to identify gene families that may have experienced gene loss, duplication, or HGT during their evolution.

Clearly, the accuracy of such approaches depends on the accuracy of the gene and species trees used in the analysis; if the topology of either (or of both) the gene and organism trees are inaccurately estimated, then the evolutionary history of the gene family may be wrongly inferred.

Assuming gene and species trees have been correctly inferred and rooted, and that no incomplete lineage sorting or intra-locus recombination has occurred (Degnan and Rosenberg, 2009; Stolzer et al., 2012), any difference between gene and organism trees can be explained by either (i) only gene loss and duplication events, (ii) only HGT and gene loss events, or (iii) a mixture of gene loss, gene duplication, and HGT (Figure 1). Note that there are many possible causes of error that could lead to inaccurate estimation of gene or species tree topologies. Such errors could lead to inaccurate conclusions concerning the frequency of gene loss, duplication, and HGT events within a gene family. These include errors in sequencing or gene prediction (Prosdocimi et al., 2012), alignment (Löytynoja and Goldman, 2008), or differences between the substitution model used to estimate the phylogeny and the true process of point mutation experienced by sequences during their evolution (Huelsenbeck and Rannala, 2004; Mar et al., 2005; Kolaczkowski and Thornton, 2008; Roure and Philippe, 2011). Furthermore, using a comparison of gene and organism trees to analyze the frequency of gene loss, duplication, and HGT events requires rooted phylogenetic trees; even with correctly estimated unrooted topologies for both gene and organism trees, errors in the inference of the position of the root of these trees will also lead to inaccurate inference of the frequencies of gene loss, duplication, and HGT (Swofford et al., 1996; Huelsenbeck et al., 2002). The inference of the position of the root of the Tree of Life (ToL) offers additional challenges due to the lack of outgroup organisms to use for comparison (Bapteste and Brochier, 2004; Lake et al., 2009).

To estimate the relative contributions of gene loss, duplication, and HGT to the taxonomic distribution and phylogenetic tree topology of a gene family requires a model of the processes, including the relative frequencies, of gene loss, gene duplication, and HGT. The accuracy with which the parameters of evolutionary models (such as phylogeny topologies, but also relative frequencies of different kinds of evolutionary change such as point substitutions between different nucleotides, but presumably also relative frequencies of gene loss, duplication, and HGT) are estimated is reduced as the evolutionary time-scale increases. Thus, we should be very cautious about the inference of ancient HGT events that may have occurred close to the origin of the eukaryotes, as is the case for the characters discussed in this review; alternative scenarios involving only vertical transmission of genetic material should also be carefully considered.

## Unexpected Character Distributions in the PVC Superphylum

Currently, relatively few characters with unexpected taxonomic distributions in PVC can be analyzed in this way, i.e., characters for which one or more of the gene families associated with the character has been identified, and for which MSAs can be built that give reasonably precise estimates of the topology of the phylogenetic tree of the gene or protein families. An example of a character that cannot be studied in this way is the presence of MC proteins in PVCs (MCs are also found also in all eukaryotes; Santarella-Mellwig et al., 2010); we have identified several relevant gene families, but the sequences of these families are so different from each other that it is impossible to confidently estimate an MSA for them, despite the clear similarity of their predicted structural features and domains (Devos, 2012). Thus, so far only three characters have been analyzed in this way: the presence and absence of (i) sterol synthesis, (ii) tubulin, and (iii) c1 transfer genes. The results of these analyses are summarized in Table 1 and in the Appendix.

## Phylogenetic Distribution of Sterol Synthesis Genes

**Figure 2:**
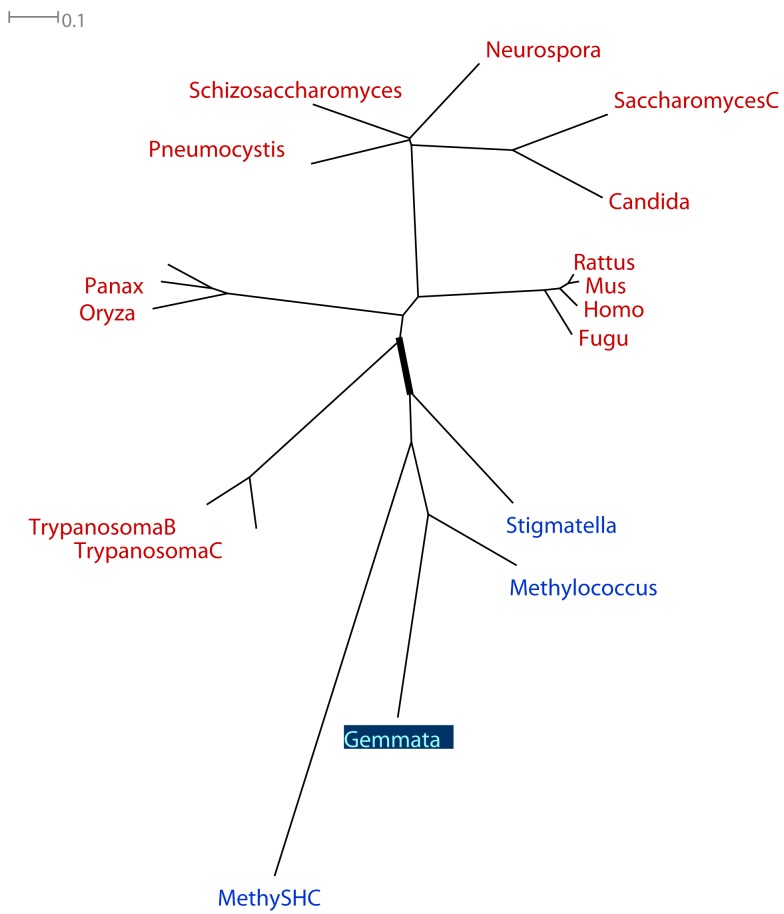
**Example phylogenetic tree considered to support a hypothesis of HGT involving PVC organisms**. Tree is adapted based on Figure 5b of an analysis of sterol synthesis by Pearson et al. (2003). The tree was drawn using all bacterial oxidosqualene cyclase (OSC)-family sequences available in the public databases at the time of the analysis. Taxon labels indicate the genus from which the sequence was sampled. The “MetylSHC” label indicates a sequence taken from the bacterium *Methylococcus capsulatus*, and is a member of the squalene–hopene cyclase (SHC) gene family, which is related to the OSC family. All eukaryotic sequences are labeled in red, all non-PVC bacterial sequences are labeled in blue, the one PVC sequence is labeled in light blue with a dark blue background. The branch that partitions all eukaryotic sequences from all bacterial sequences is drawn thicker than all other branches in the tree.

An analysis of the taxonomic distribution of sterol synthesis genes in eukaryotes, planctomycetes, and other bacteria illustrates many of the issues discussed here (Pearson et al., 2003). Sterol synthesis is found in almost all eukaryotes, but in only a few bacteria. Pearson et al. present phylogenetic trees of the only two genes involved in sterol synthesis in PVCs (Appendix). Both trees include several (more than 10) eukaryotic sequences, at least one non-PVC bacterial sequence, and one PVC sequence. Both trees contain an internal branch that partitions all eukaryotic sequences from all bacterial sequences (Figure 2).

Because the estimated gene tree corresponded to the canonical “16S rRNA phylogeny” (with three monophyletic domains of eukaryotes, bacteria, and archaea), the authors concluded that “a recent lateral gene transfer from higher-order eukaryotes to bacteria is unlikely. The bacterial sterol biosynthesis genes are not closely related to genes found in any extant group of eukaryotes, and the bacterial gene trees are parsimonious with respect to 16S rRNA phylogeny.” Alternatives to the 16S rRNA phylogeny for the ToL were not considered.

However, due to the absence of members of these gene families from most bacterial genomes, the authors concluded that the observed taxonomic distribution of these families is best explained by an ancient HGT between bacteria and eukaryotes. Alternative explanations involving no HGT, i.e., only vertical genetic transfer and gene loss, were considered less likely than the HGT scenario. This is because, in the implicit model of gene family evolution used by the authors, one HGT is considered more likely than the large (but unspecified) number of gene losses inferred by alternative scenarios.

Another reason why non-HGT scenarios are considered unlikely in this, and other similar, analyses, is that they require the inference of a change in the rate of gene loss along different lineages. More specifically, they require the inference of an initial period in which gene loss is high, followed by a long period in which the gene families are retained by a small number of organisms, i.e., lineages in which the rate of gene loss is much reduced. Thus, one (or a few) HGT events are considered more likely than a relatively more “complex” scenario in which the rate of gene loss (and other events) varies (i.e., is time-heterogeneous) across the phylogeny.

Similar conclusions were reached, for similar reasons, by many analyses of PVC characters with unexpected character distributions (Table 1 and Appendix.).

## Frequent Inference of HGT to Account for Unexpected Character Distributions; A Need for Caution

In many cases, these analyses have concluded that the most likely explanation for the phylogenetic distribution of these gene or protein families involves one or more ancient HGT events. In this article, we review these previous studies, focusing in particular on two aspects of their results and conclusions. Firstly, we highlight, as reported by the authors, that the divergence of PVC and eukaryotic/archaeal members of these families are ancient events. Secondly, most of these studies conclude that one or more HGT events are likely to have occurred during the evolution of these families; we discuss three issues that make us cautious about making such inferences, i.e., that (i) it is difficult to obtain accurate inference of phylogenetic tree topologies for single gene families that diverged over the time-scales involved in these analyses (ii) inference of HGT also requires an estimate of the species tree for the organisms involved in the analysis, which is also difficult, and (iii) the need for increased use and further development of probabilistic models of the different kinds of genetic events that can shape the taxonomic distribution of gene families (i.e., gene duplication, vertical inheritance, gene loss, and HGT).

Therefore, we encourage caution in the inference of ancient HGT events to explain unexpected character distributions, and look forward to the further development of probabilistic models for comparing HGT and non-HGT-based models. Additionally, we think that it is important for such analyses to consider alternatives to the three-domain 16sRNA-based ToL when assessing the evidence for HGT in general. In the case of the PVC characters, alternatives include the possibility that the engulfment of a thaumarchaeon by a PVC bacterium was involved in the origin of the eukaryotes (Forterre, 2010), a stepwise vertical evolution of archaea and eukaryotes from a PVC common ancestor (Devos and Reynaud, 2010; Reynaud and Devos, 2011), or the reductive evolution of Planctomycetes from a complex proto-eukaryote-like last universal common ancestor (Fuerst and Sagulenko, 2011, 2012). Many of these issues are generally relevant to inference of HGT events, not just in the context of unexpected character distributions involving PVCs.

## Accurate Inference of Distant Phylogenetic Relationships is Hard

Estimating patterns of genetic information transfer, i.e., phylogenetic tree topologies, that include ancient lineage divergences is difficult (Gribaldo and Philippe, 2002; Delsuc et al., 2005). In particular, over large time-scales, we expect variation in the (nucleotide or amino acid) substitution processes acting on different branches of the phylogeny, while most phylogenetic inference uses homogeneous substitution models that assume the same process acts on all branches of the tree (Roure and Philippe, 2011). Model misspecification of this kind has been found in a range of different sequence datasets, and has been shown to potentially lead to systematic error in phylogeny reconstruction (Shavit Grievink et al., 2010). Such errors can lead to the estimation of branches with high support values (i.e., with low sampling error) that are not present in the true phylogeny.

The size of the dataset (i.e., the number of MSA alignment columns) used to estimate phylogenies for individual gene families is clearly restricted by the size of the gene being analyzed. Organism phylogenies are typically estimated from much larger data sets obtained by combining data from many different genes. Thus, phylogenies estimated for individual genes typically experience increased sampling error compared to organism trees (Jeffroy et al., 2006; Rokas and Carroll, 2006; Castresana, 2007). The phylogenetic trees used to investigate the role of HGT in establishing unexpected taxonomic distributions of characters in PVCs all involve ancient lineage divergences, typically around the time of the origin of the eukaryotes or earlier. Thus, we should be aware that there could be significant undetected errors in both the gene and organism phylogenies used in these analyses. In particular, some of the gene trees used in these analyses include many long branches clustered together within the tree (Pearson et al., 2003; Bauer et al., 2004; Chistoserdova et al., 2004), a feature that could be the result of systematic errors often referred to as “long branch attraction” (Huelsenbeck, 1997; Anderson and Swofford, 2004; Bergsten, 2005).

## Extensive Disagreement on Many Features of the Tree of Life

Many of the analyses reviewed in this paper assume that the three-domain ToL estimated from early 16S rRNA analyses and other later studies (Woese et al., 1990) is correct, and do not take into account any alternative ToLs. However, the organism phylogenies used to infer HGT in the evolutionary history of PVC gene members may contain potentially major topological errors. One way of highlighting this is to consider current disagreements concerning the ToL. In recent years, several alternatives to the classical three-domain ToL have been proposed, for example the “eocyte” hypothesis in which the archaea are not monophyletic (Cox et al., 2008; Foster et al., 2009), and many alternatives have been proposed for other features of the ToL, such as the interrelationship between eukaryotes and archaea, and the relationships between the major eukaryotic groups (Burki et al., 2007; Hampl et al., 2009; Roger and Simpson, 2009; Desmond et al., 2010). In addition to this disagreement and discussion of fundamental features of the ToL, it has also been shown that the phylogenetic signal present in “universal” proteins (i.e., which are found in the majority of cellular organisms) are not sufficient to resolve with confidence the topology of the ToL (Desmond et al., 2010). A recent analysis of the origin of land plants (Laurin-Lemay et al., 2012) nicely illustrates many factors that can make it difficult or impossible to estimate a true organismal phylogeny, including closely spaced speciation events, incomplete lineage sorting, gene duplications, and HGTs. Of particular relevance to analyses involving genes found in the PVC superphylum is the ambiguity of the phylogenetic position of the group (Stackebrandt et al., 1984; Janssen et al., 1997; Ward et al., 2000; Jenkins and Fuerst, 2001; Brochier and Philippe, 2002; Di Giulio, 2003; Fieseler et al., 2004; Teeling et al., 2004), further highlighting the uncertainty associated with the topology of the ToL.

Hopefully, in the future, improved taxon sampling and the use of more sophisticated models of character evolution may lead to a more accurate estimate of the ToL (Philippe et al., 2011).

Thus, given the range of alternatives proposed for many different parts of the ToL, it is clearly important to be cautious when using it to investigate possible HGT events. In particular, where alternative hypotheses for the topology of regions of the ToL may lead to different conclusions about the occurrence of HGT, then such analyses should be carried out using all plausible alternative ToLs.

## The Relative Probability of Gene Loss, Gene Duplication, and HGT are Poorly Understood

A key component of HGT inference using the comparison of gene and species trees is a model, whether implicit or explicit, of the processes of vertical and horizontal genetic transmission, as well as of the relative probability of gene duplication and loss.

The use of explicit probabilistic models of these processes enables the use of formal statistical tests of whether the observed data (gene and organism trees) better fit a scenario with or without HGT. However, relatively little attention has been given to models of this kind, and in most cases, the inference of HGT is considered outside such an explicit model-testing framework. Part of the reason for this is perhaps that researchers are cautious about explicitly building models for a process (HGT) which is relatively poorly understood, in terms of factors likely to influence variation in the rate at which it occurs such as gene size, features, and degree of divergence of accepting and donating genomes, and other factors (Sorek et al., 2007; Boto, 2010). Similar uncertainty surrounds the dynamics of the processes of gene duplication and gene loss. For example, there is strong evidence that some gene families in parasitic bacteria experience an increased rate of gene loss, analogous to the heterotachy observed in the process of point mutations in sequence evolution (Kolaczkowski and Thornton, 2008), i.e., where the rate of an evolutionary process varies along different lineages of a phylogeny.

However, despite the problems associated with the use of explicit models of such processes, we feel that it would be good if assertions of HGT (or non-HGT) were carried out in the context of exploration of some of the existing models of this kind (Thiergart et al., 2012). Explicitly declaring the sets of assumptions being made when HGT is (or is not) being inferred, i.e., by using explicit probabilistic models of this kind, would ease the process of identifying and discussing the central assumptions lying with the inferences. Hopefully, in the future, a better understanding of the processes of gene family evolution will aid the development of more sophisticated and accurate models of these processes, leading to a wider application of methods of this kind.

## Conclusion

As described above, given:

1) the inherent difficulty of accurately estimating ancient phylogenetic relationships,
2) our uncertainty concerning the topology of many parts of the ToL, in particular the relationship between the three domains of life and the position of the PVC within the bacteria, and
3) the need for wider use of and further development of methods used to compare HGT with non-HGT scenarios,

it is important to be cautious about inferring the occurrence of ancient HGT to account for unexpected distributions of characters and gene families. Ideally, we feel such inferences should be made in the context of testing whether or not HGT is supported using a range of different explicit models, while also taking into account the uncertainty and proposed alternatives of the trees (both organism and gene trees). Thus the HGT origin of those PVC features is still not established. This uncertainty highlights the importance of taking into account new and alternative hypotheses and ideas in analyses of this kind (Devos and Reynaud, 2010; Forterre, 2010; Reynaud and Devos, 2011; Fuerst and Sagulenko, 2012). In this context, discussions about the evolutionary origins of some of the characters found in PVCs echo the initial reaction to the platypus by European scientists at the beginning of the nineteenth century; with its mosaic of characters not previously seen together in the same organism (including, amongst others, a beak like a duck, eggs similar to those of reptiles or birds, together with the fur and milk production found in other of mammals) it was initially assumed to be a hoax. In contrast to this initial reaction, however, the discovery of the platypus provided a rich source of ideas and understanding about the history and process of both mammalian and non-mammalian evolution, along with better understanding of the connections between these taxonomic groups. In a similar way, with its surprising and unexpected combination of characters, the PVC superphylum might help reveal new and unexpected links and similarities between bacteria and other cell types, including our own.

## Conflict of Interest Statement

The authors declare that the research was conducted in the absence of any commercial or financial relationships that could be construed as a potential conflict of interest.

## Appendix

### The PVC superphylum

The Planctomycetes-Verrucomicrobia-Chlamydiae (PVC) superphylum is an assemblage of bacterial phyla which is consistently recovered as a monophyletic group in 16S rRNA trees estimated using a range of different phylogeny estimation methods (Wagner and Horn, 2006). It includes the Planctomycetes, a group of ubiquitous bacteria found in soil, fresh water, the oceans, and most other locations in which microbial biodiversity has been analyzed (Fuerst and Sagulenko, 2011), the Verrucomicrobia, which includes a small number of species isolated from fresh water, soil environments, and human feces, the Chlamydiae, a bacterial phylum whose members are obligate intracellular pathogens, the Poribacteria, found so far only in sponges, the Lentisphaerae, which includes marine microorganisms and terrestrial gut microbiota, and the OP3 candidate phyla which contain no cultured relatives, along with several other groups. The inference that this group of organisms form a natural group was initially unexpected. However, since then it has been recovered by a range of further analysis, and has become increasingly accepted as a valid taxonomic grouping (Wagner and Horn, 2006; Pol et al., 2007; Hou et al., 2008; Pilhofer et al., 2008; Kamneva et al., 2010).

#### Sterol synthesis pathway

Sterol synthesis starts with the oxygenation of a squalene precursor by the enzyme squalene monooxygenase (SQMO), followed by the cyclization of the epoxide to lanosterol or cycloartenol by the oxidosqualene cyclase (OSC) protein. In most organisms capable of sterol synthesis, these simple sterols are subsequently modified. SQMO and OSC genes have so far been described in only three bacterial phyla, γ-proteobacteria (in *Methylococcus capsulatus*), δ-proteobacteria (in *Stigmatella aurantica* and *Plesiocystis pacifica*), and Planctomycetes (in *Gemmata obscuriglobus*), but are prevalent in eukaryotic organisms. Four phylogenetic analyses have been published for this pair of genes (Pearson et al., 2003; Chen et al., 2007; Desmond and Gribaldo, 2009).

The phylogenies estimated in the first of these analyses (Pearson et al., 2003) contained a branch that partitioned all bacterial sequences from all eukaryotic sequences (i.e. the tree contained a bacteria-only and a eukaryote-only clan, see Figure 2 in the main text of this current article), and thus concluded that “a recent lateral gene transfer from higher-order eukaryotes to bacteria is unlikely. The bacterial sterol biosynthesis genes are not closely related to genes found in any extant group of eukaryotes, and the SQMO and OSC trees are parsimonious with respect to 16S rRNA phylogeny.” Additionally, the authors analyzed the sterols produced by *G. obscuriglobus*, their simple structure suggesting that this genus might retain ancient remnants of the sterol biosynthetic pathway. The authors however proposed that an ancient HGT between bacteria and ancient eukaryotes best explained the observed phylogenies, as alternative non-HGT scenarios were considered to be more unlikely because they require the inference of a large number of gene losses.

Similar conclusions were described in the second analysis (Chen et al., 2007). This phylogenetic analysis of only the OSC genes identified a bacterial and a eukaryotic-only clan. Again, the authors suggested that “the (HGT of sterol biosynthesis) genes from eukaryotes to bacteria occurred soon after the divergence of eukaryotes and bacteria.”

In the third analysis (Desmond and Gribaldo, 2009), it was noted that the bacterial SQMO homologs “branch basally, but do not appear to be more closely related to eukaryotic (genes) than they are to other bacterial monooxygenases, and they share no specific sequence signature with” the eukaryotic genes. The other bacterial genes, OSC “appear to be more closely related to their eukaryotic homologs, indicating a specific evolutionary relationship.” In this case, only one of the four bacterial OSC homologs branched within the eukaryotes, suggesting that it was obtained via HGT. However, the other bacterial OSC homologs branched basally, “which could be interpreted in favor of a hypothesis where the eukaryotic gene originated from bacteria.” It was then concluded that the gene families having bacterial homologs have likely been “recruited from preexisting enzymes in parallel to the emergence of the sterol pathway in the lineage leading to the Last Eukaryotic Common Ancestor.” The authors also put forward the “possibility that (SQMO) in these bacteria does not derive from HGT from eukaryotes.”

In the fourth analysis, the authors investigated the *G. obscuriglobus* OSC (Frickey and Kannenberg, 2009). They reported that the planctomycete sequence is more similar to the eukaryotic one but that it was “too close to the base of the phylogenetic tree to make any relevant statement regarding their evolutionary history,” specifically about potential HGT involvement. They continue by stating that the *G. obscuriglobus* sequence “is too basal in the eukaryotic group of OSCs to be taken further into account as potential HGTs.” Interestingly, they concluded that “based on our bioinformatics analysis it seems plausible that these genes may have been laterally transferred from eukaryotes to bacteria; however, as aforementioned, these candidates are too close to the ‘phylogentic base’ of the eukaryotic group to make a convincing cases for HGT.”

Thus, there is some difference of opinion amongst the various analyses concerning how likely it is that ancient HGT played a role in the evolutionary history of the families, a recurrent pattern throughout the analysis of the PVC characters.

#### Tubulin

One of the major structural features that separates the eukaryotes from bacteria and archaea is the presence of an internal cytoskeleton composed principally of tubulin. Tubulin genes are present in all eukaryotes and are almost entirely absent from the bacteria and archaea. The only bacterial exceptions being the bacterial Verrucomicrobia genus *Prosthecobacter*, where two copies of tubulin have been detected in some species. In the initial study (Jenkins et al., 2002), the conclusion drawn from the results of their phylogenetic analyses was that “the *Prosthecobacter* tubulins are quite divergent and do not support recent (HGT) of the genes from a eukaryote.” It was additionally concluded that “the bacterial tubulins are ancestral to eukaryotic tubulins” and that this could also be explained “in terms of a shared ancestry between the two groups or a gene transfer from an ancestor of the Verrucomicrobia to a protoeukaryotic organism, before the radiation of extant eukaryotes.” A recent phylogenetic analysis (Pilhofer et al., 2011) similarly failed to detect any well-supported phylogenetic grouping between the bacterial tubulins with any of the eukaryotic tubulin subfamilies, i.e., the bacterial genes branch basally relative to the eukaryotic ones. The authors concluded that the presence of tubulin in bacteria is not the result of a transfer from a modern eukaryote, and thus that the bacterial tubulins “should therefore be considered as two novel tubulin subfamilies, derived not from any particular modern subfamily but instead directly from ancient tubulins and that rather than being derived from modern eukaryotic tubulin, the *Prosthecobacter* tubulins arose from early tubulin intermediates.”

#### C1 transfer genes

The case of C1 transfer genes is more complex. Two alternative bioconversions are responsible for maintaining the global methane balance on Earth. Most methane producers (methanogens) are archaea, and most methane consumers (methanotrophs) are either proteobacteria (aerobic) or archaea (anaerobic). Once again the exceptions are found in PVC members, some Planctomycetes are methanogens, while some Verrucomicrobia are methylotrophs.

The two initial analyses of planctomycete methanogen genes lead to contradictory conclusions. In the first analysis (Chistoserdova et al., 2004), it was observed that “phylogenetic analysis places the planctomycete sequences as distantly from their archaeal counterparts as from their proteobacterial counterparts.” It was concluded that “Planctomycetes sequences diverge significantly from their proteobacterial counterparts and occupy an intermediate phylogenetic position between archaea and proteobacteria” (Chistoserdova et al., 2004). A concomitant hypothesis based on almost the same set of sequences (Bauer et al., 2004) reached a different conclusion, i.e., that the most likely scenario for the evolution of these gene families involved ancient HGT events. The authors of this study acknowledge that the available data does not exclude the possibility that the evolution of these gene families did not involve any HGT.

Concerning the alternative reaction, methylotrophy, a central enzyme family in the bacterial methylotrophs is the Mtd family, that is unrelated to the archaeal counterpart. MtdB is found in methylotrophs using the pathway that involves tetrahydromethanopterin (H_4_MPT) as a cofactor, while the paralog, MtdA, is so far only found in methylotrophs employing the serine cycle for formaldehyde assimilation. A third ortholog, MtdC, is found only in planctomycetes and an uncharacterized microbe. Phylogenetically, the planctomycete MtdC falls into a distinct group that is clearly separated from both MtdA and MtdB enzymes (Vorholt et al., 2005). Again, an ancestral role of Planctomycete MtdC protein with respect to both MtdA and MtdB was speculated based on broader substrate specificity. A concomitant phylogenetic analysis of the H_4_MPT pathway proteins found the genes to branch basally to the proteobacterial ones (Kalyuzhnaya et al., 2005). Based on the comparison of the protein and organismal trees, the authors concluded that the gene history possibly involved HGT between Proteobacteria and other phyla, including planctomycetes.

Analysis of the Verrucomicrobia (methanotrophs) proteins were more conclusive, indicating an ancient divergence from proteobacteria, the other methanotrophe bacteria (Op den Camp et al., 2009). A subsequent phylogenetic analysis of the genes encoding subunits of the membrane-bound methane monooxygenase in the verrucomicrobia *Methylokorus infernorum* “placed them into a distinct cluster from proteobacterial homologs. This indicates an ancient divergence of Verrucomicrobia and Proteobacteria methanotrophs rather than a recent horizontal gene transfer of methanotrophic ability” (Dunfield et al., 2007).

Carbon fixation in methanotrophs uses the ribulose-1,5-bisphosphate carboxylase/oxygenase (RuBisCO) enzyme in the Calvin–Benson–Bassham (CBB) cycle. Phylogenetic analysis suggested that the verrucomicrobial methanotrophs RuBisCO represents a new type of enzyme, with the Verrucomicrobia enzymes forming a distinct group separated from the other prokaryotic RuBisCO (Khadem et al., 2011). As with sterol and tubulin, it was concluded that “the streamlined metabolism of verrucomicrobial methanotrophs may be reminiscent of the metabolism of primordial methanotrophs” (Chistoserdova, 2011). No HGT appeared to have been involved. Similarly to tubulin, an ancient relationship is observed for the PVC C1 transfer genes.

In conclusion, as described in the main text, given:

1) the inherent difficulty of accurately estimating ancient phylogenetic relationships,
2) our uncertainty concerning the topology of many parts of the ToL, in particular the relationship between the three domains of life and the position of the PVC within the bacteria, and
3) the need for wider use of and further development of methods used to compare HGT with non-HGT scenarios,

it is important to be cautious about inferring the occurrence of ancient HGT to account for unexpected distributions of characters and gene families. Ideally, we feel such inferences should be made in the context of testing whether or not HGT is supported using a range of different explicit models, while also taking into account the uncertainty and proposed alternatives of the trees (both organism and gene trees). Thus, in our opinion, the HGT origin of these PVC features remains to be established. This uncertainty highlights the importance of taking into account new and alternative hypotheses and ideas in analyses of this kind (Devos and Reynaud, 2010; Forterre, 2010; Reynaud and Devos, 2011; Fuerst and Sagulenko, 2012).

## Abbreviations

HGT, horizontal gene transfer; MC, membrane coat protein; MSA, multiple sequence alignment; PVC, planctomycetes-verrucomicrobiae-chlamydiae.

## References

[B67] Chen L.-L. Wang G.-Z. Zhang H.-Y. (2007). Sterol biosynthesis and prokaryotes-to-eukaryotes evolution. Biochem. Biophys. Res. Commun. 363, 885–888 10.1016/j.bbrc.2007.09.093 17923113

[B68] Chistoserdova L. (2011). Modularity of methylotrophy, revisited. Environ. Microbiol. 13, 2603–2622 10.1111/j.1462-2920.2011.02464.x 21443740

[B69] Desmond E. Gribaldo S. (2009). Phylogenomics of sterol synthesis: insights into the origin, evolution, and diversity of a key eukaryotic feature. Genome Biol. Evol. 1, 364–381 10.1093/gbe/evp036 20333205 PMC2817430

[B70] Dunfield P. F. Yuryev A. Senin P. Smirnova A. V. Stott M. B. Hou S. (2007). Methane oxidation by an extremely acidophilic bacterium of the phylum Verrucomicrobia. Nature 450, 879–882 10.1038/nature06411 18004300

[B71] Frickey T. Kannenberg E. (2009). Phylogenetic analysis of the triterpene cyclase protein family in prokaryotes and eukaryotes suggests bidirectional lateral gene transfer. Environ. Microbiol. 11, 1224–1241 10.1111/j.1462-2920.2008.01851.x 19207562

[B72] Jenkins C. Samudrala R. Anderson I. Hedlund B. P. Petroni G. Michailova N. (2002). Genes for the cytoskeletal protein tubulin in the bacterial genus Prosthecobacter. Proc. Natl. Acad. Sci. U.S.A. 99, 17049–17054 10.1073/pnas.012516899 12486237 PMC139267

[B73] Kalyuzhnaya M. G. Korotkova N. Crowther G. Marx C. J. Lidstrom M. E. Chistoserdova L. (2005). Analysis of gene islands involved in methanopterin-linked C1 transfer reactions reveals new functions and provides evolutionary insights. J. Bacteriol. 187, 4607–4614 10.1128/JB.187.21.7511-7517.2005 15968072 PMC1151760

[B74] Khadem A. F. Pol A. Wieczorek A. Mohammadi S. S. Francoijs K.-J. Stunnenberg H. G. (2011). Autotrophic methanotrophy in verrucomicrobia: Methylacidiphilum fumariolicum SolV uses the Calvin–Benson–Bassham cycle for carbon dioxide fixation. J. Bacteriol. 193, 4438–4446 10.1128/JB.00407-11 21725016 PMC3165502

[B75] Op den Camp H. J. M. Islam T. Stott M. B. Harhangi H. R. Hynes A. Schouten S. (2009). Environmental, genomic and taxonomic perspectives on methanotrophic Verrucomicrobia. Environ. Microbiol. Rep. 1, 293–306 10.1111/j.1758-2229.2009.00022.x 23765882

[B76] Pilhofer M. Ladinsky M. S. McDowall A. W. Petroni G. Jensen G. J. (2011). Microtubules in bacteria: ancient tubulins build a five-protofilament homolog of the eukaryotic cytoskeleton. PLoS Biol. 9, e1001213 10.1371/journal.pbio.1001213 22162949 PMC3232192

[B77] Vorholt J. A. Kalyuzhnaya M. G. Hagemeier C. H. Lidstrom M. E. Chistoserdova L. (2005). MtdC, a novel class of methylene tetrahydromethanopterin dehydrogenases. J. Bacteriol. 187, 6069–6074 10.1128/JB.187.17.6069-6074.2005 16109948 PMC1196156

